# A machine learning classifier using 33 host immune response mRNAs accurately distinguishes viral and non-viral acute respiratory illnesses in nasal swab samples

**DOI:** 10.1186/s13073-023-01216-0

**Published:** 2023-08-28

**Authors:** Rushika Pandya, Yudong D. He, Timothy E. Sweeney, Yehudit Hasin-Brumshtein, Purvesh Khatri

**Affiliations:** 1 Inflammatix Inc., CA 94085 Sunnyvale, USA; 2 Institute for Immunity, Transplantation and Infection, School of Medicine, Stanford University, Stanford, CA 94305 USA; 3 Allen Institute of Immunology, Seattle, WA USA; 4 Department of Medicine, Center for Biomedical Informatics Research, School of Medicine, Stanford University, Stanford, CA 94305 USA

## Abstract

**Background:**

Viral acute respiratory illnesses (viral ARIs) contribute significantly to human morbidity and mortality worldwide, but their successful treatment requires timely diagnosis of viral etiology, which is complicated by overlap in clinical presentation with the non-viral ARIs. Multiple pandemics in the twenty-first century to date have further highlighted the unmet need for effective monitoring of clinically relevant emerging viruses. Recent studies have identified conserved host response to viral infections in the blood.

**Methods:**

We hypothesize that a similarly conserved host response in nasal samples can be utilized for diagnosis and to rule out viral infection in symptomatic patients when current diagnostic tests are negative. Using a multi-cohort analysis framework, we analyzed 1555 nasal samples across 10 independent cohorts dividing them into training and validation.

**Results:**

Using six of the datasets for training, we identified 119 genes that are consistently differentially expressed in viral ARI patients (*N* = 236) compared to healthy controls (*N* = 146) and further down-selected 33 genes for classifier development. The resulting locked logistic regression-based classifier using the 33-mRNAs had AUC of 0.94 and 0.89 in the six training and four validation datasets, respectively. Furthermore, we found that although trained on healthy controls only, in the four validation datasets, the 33-mRNA classifier distinguished viral ARI from both healthy or non-viral ARI samples with > 80% specificity and sensitivity, irrespective of age, viral type, and viral load. Single-cell RNA-sequencing data showed that the 33-mRNA signature is dominated by macrophages and neutrophils in nasal samples.

**Conclusion:**

This proof-of-concept signature has potential to be adapted as a clinical point-of-care test (‘RespVerity’) to improve the diagnosis of viral ARIs.

**Supplementary Information:**

The online version contains supplementary material available at 10.1186/s13073-023-01216-0.

## Background

Acute respiratory illnesses (ARI) significantly contribute to human mortality. Even prior to the COVID-19 pandemic, ARIs caused more than 2 million deaths annually and were the sixth major cause of mortality for all ages [1, 2]. Viral infections are a common cause of ARIs and require different treatment than non-viral ARIs. Typically, nasal samples of ARI patients are routinely screened for a predetermined set of common viruses [3, 4]. However, the sensitivity of nasal swab-based diagnostic tests varies widely as has been demonstrated repeatedly for multiple viruses including influenza [5] and SARS-CoV-2 [6–9], which allow ruling in, but not ruling out viral infections. Furthermore, the multiple pandemics of the twenty-first century, including the ongoing COVID-19 pandemic, have demonstrated that the current practice of using a predetermined set of viruses severely limits our ability to detect clinically relevant emerging pathogens in a timely manner [5]. Although metagenomic sequencing can identify novel viruses in pooled human samples [8–11], identification of a pathogen in human meta-samples does not directly translate into a health risk. There is an unmet need for novel diagnostics that can enable ruling out viral infection with higher confidence and identify patients with emerging viral infections of clinical relevance.

Recent studies have repeatedly highlighted the utility of host response-based diagnostics in addressing these challenges, and multiple host response-based tests are in late development [12–15]. Importantly, the host response to viral infection in peripheral blood is conserved and can distinguish it from other inflammatory conditions. For example, we have demonstrated that host response-based gene signature in peripheral blood, identified using known respiratory viral infections [16], is also conserved in emerging viruses, including SARS-CoV-2, chikungunya, and Ebola, and is associated with the severity of viral infections [17]. Similarly, Mick et al. described a conserved host response to viral infection in nasopharyngeal/oropharyngeal swabs that is distinct from patients with other ARIs [18]. A recent pathogen surveillance and detection study demonstrated that in symptomatic patients, who tested negative for a panel of respiratory viruses using multiplex PCR but had higher levels of cytokines in nasal samples, host response in nasopharyngeal swab identified clinically relevant infection in 75% of samples, of which > 35% of patients had acute viral infection [19]. These results suggest that similar to blood-based host response diagnostics, conserved host response to viral infections in nasal samples can also be utilized for diagnosis and to rule in or rule out viral infection in symptomatic patients when targeted pathogen-diagnostic tests are negative. Nasal samples also have certain advantages over blood samples. First, nasal samples are easy to obtain and routinely obtained in clinical practice. Second, measuring host response in the respiratory tract could enable earlier detection of viral infection.

Several studies have profiled host response in nasal samples to identify gene sets for diagnosis of viral infection. However, none have been shown to generalize to broad populations [20–25]. One of the important factors limiting the translation to clinical practice is the lack of heterogeneity within cohorts from which these genes were identified, which do not generalize to the real-world patient population. Using a multi-cohort analysis framework [26, 27], we have repeatedly demonstrated that leveraging biological, technical, and clinical heterogeneity across diverse cohorts can identify robust host response changes in patients with infections that can be translated to clinical tests [14, 28]. For instance, we have successfully applied multi-cohort analysis to develop a clinically useful blood-based classifier that reliably distinguishes between viral and bacterial infections [29–31].

We hypothesized that multi-cohort analysis of whole transcriptome profiles from nasal samples of patients with or without viral infection can identify a robust nasal host response gene expression signature broadly conserved across populations that could be translated to clinical use. We applied multi-cohort analysis to transcriptome profiles of 1555 nasal samples available across 10 public datasets. We identify a conserved host response gene signature that distinguishes viral ARI samples from either healthy controls or non-viral ARIs with high accuracy. We found that the host response is robust to demographic and clinical variables such as age, virus type, or viral load and believe that these results provide a solid foundation for diagnostic test development.

## Methods

### Data collection

We conducted a systematic search in Gene Expression Omnibus (GEO) for datasets with transcriptomic data using keywords respiratory viral infections, viral ARI, and respiratory viral infections in November 2021. After manual curation of all datasets, we identified 10 datasets that met the inclusion criteria (Table 1): included nasal samples from subjects with viral ARI and control subjects, and control samples were derived from healthy donors or subjects recovered from ARI as well as subjects with non-viral respiratory illness.

**Table 1:**
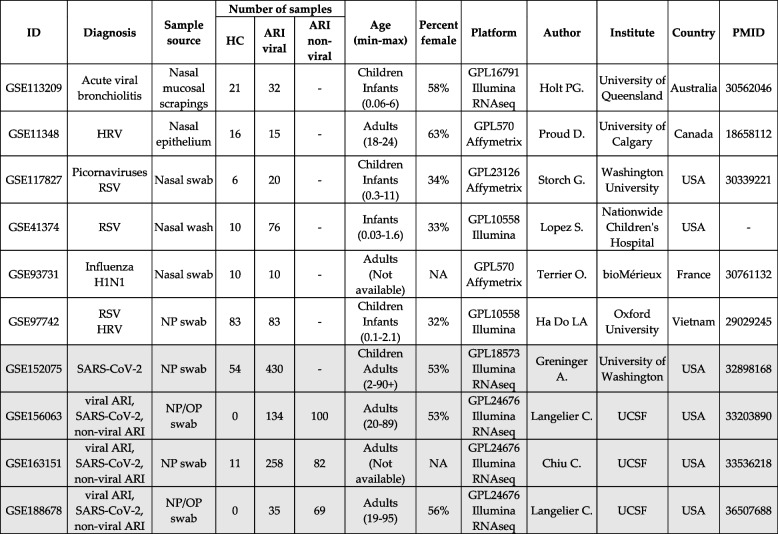
Gene expression studies for viral ARI with nasal samples

The first 6 studies were used to identify a gene signature to distinguish viral ARI samples from controls; the last four studies highlighted in grey were used for validation. Age column includes group and age range in years if available

*NP* nasopharyngeal, *OP* oropharyngeal, *HC* healthy controls

### Description of datasets

#### GSE113209

Nasal mucosal scrapings (NMS) from infants (< 18 months) and children (1.5–5 years) during acute viral bronchitis and post-convalescence. Immune response patterns were profiled by multiplex analysis of plasma cytokines, flow cytometry, and transcriptomics (RNA-Seq). Study was conducted in Australia [32].

#### GSE11348

Gene expression changes evaluated by microarray in nasal scrapings of adult subjects inoculated with rhinovirus or sham control at 8 and 48 h after inoculation. Study was conducted in the USA [21].

#### GSE117827

Comparison of host transcriptomic response by microarray in nasal and blood samples of children with viral infections and various levels of symptoms (acute respiratory syncytial virus (RSV) infection, symptomatic non-RSV respiratory virus infection, asymptomatic rhinovirus infection, and virus-negative asymptomatic controls. Study was conducted in the USA [22].

#### GSE41374

Microarray-based gene expression of nasal wash samples collected from infants infected with RSV within 48 h of hospital admission and 10 healthy controls [33].

#### GSE93731

Microarray-based transcriptomic signatures from nasal swabs collected of patients with H1N1 influenza infection. Samples were collected either at inclusion (before any antiviral treatment, infected status) or 3 months after recovery (cured status). Study was conducted in France [34].

#### GSE97742

Microarray-based transcriptional profiles of nasopharyngeal swabs collected from children hospitalized with lower respiratory tract infections and diagnosed with either RSV or rhinovirus. Study conducted in Vietnam [23].

#### GSE152075

Bulk RNA-seq transcriptomic profiles of nasopharyngeal swabs collected from patients with SARS-CoV-2 infection and healthy controls. Study was conducted in the USA and includes patients with variable infection status, viral load, age, and sex [35].

#### GSE156063

Bulk RNAseq transcriptomic profiles of upper airway samples collected from children and adults with viral or non-viral acute respiratory illnesses (ARIs). Study conducted in the USA [18].

#### GSE163151

Bulk RNAseq transcriptomic profiles of nasopharyngeal swabs from adults with viral and non-viral acute respiratory infections and donor controls [36].

#### GSE188678

Bulk RNAseq transcriptomic profiles of upper airway samples collected from children and adults with viral or non-viral acute respiratory illnesses (ARIs). Study conducted in the USA [37].

### Gene expression normalization

We processed the microarray datasets before analysis. Specifically, we downloaded original data files (.CEL) and normalized all data using the Robust Multichip Average (RMA) method from the affy R package [38]. Similarly, we processed the RNA-Seq datasets using our pipeline described previously [39]. In brief, we used FASTQC to assess multiple quality control metrics [40]. We used STAR aligner (version 2.7.3a) [41] to map the reads to the human reference genome and transcriptome (versions GRCh38 and GENCODE v32 primary assembly GTF, respectively) [41, 42]. We generated the read counts for all the samples using STAR. Finally, we normalized the count data using Voom transform. Specifically, low-expressed genes were filtered using the following cutoff: max counts per million (CPM) less than 5 across all samples from a dataset. The voom method (limma R package) was then used to transform counts into normalized log2-CPM. Two studies, GSE188678 and GSE156063, were published by the same authors. Therefore, we used Pearson correlation of gene expression as well as matching internal identifier ("Sample_title"), age, and sex provided in the GEO submission for each sample from GSE188678 with all samples from GSE156063 to investigate whether any samples were overlapping between the two studies. We found 214 out of 318 samples in GSE188678 were also included in GSE156063 as they had the same age and sex reported and had almost perfect correlation for COVID-19 PCR results (*r* = 0.994). Therefore, we only used the remaining 104 samples from GSE188678 in our analysis.

### Multi-cohort analysis

We downloaded the 10 transcriptomic datasets from GEO together with phenotypic data. We used 6 datasets for discovery and reserved 4 datasets for validation. We performed a well-established multi-cohort analysis on the 6 discovery datasets using the MetaIntegrator package (v2.1.1) in R [27]. Briefly, we calculated the effect size (ES) for each gene within a study between cases (viral ARI samples) and controls as Hedges’ *g*. The pooled ES across all datasets was computed using DerSimonian and Laird random-effects model. After summarizing the effect size, *p*-values across all genes were corrected for multiple testing based on Benjamini–Hochberg’s false discovery rate (FDR). We used Fisher’s sum of logs method for combining *p*-values across datasets. Log-sum of *p*-values that each gene is over- or under-regulated was computed along with corresponding *p*-values. Again, we used the Benjamini–Hochberg method to correct for multiple testing across all genes. Finally, we used an absolute ES threshold of ≥ 0.6 in conjunction with FDR ≤ 0.1, and the availability of gene measurement across all 10 datasets to filter genes for the discovery datasets.

### Guided forward search

To reduce the number of genes used for the final model, we used the forward search approach [15]. Briefly, forward search is an iterative process where the algorithm starts with the gene with the highest single ES and keeps adding genes to the model one by one based on positive contribution to the model discriminatory power. Forward search often results in a small set of several genes that retains the performance of the entire set. However, one of the pitfalls of any forward search is the dependence on starting point and potential overfitting to the particular set of training datasets. Therefore, here, we explored a modified forward search where we use multiple starting points—specifically we chose 12 genes (top 10%) with maximum absolute pooled effect size in training sets and used each one of those in a separate forward search run. We then used all 33 mRNAs that were identified in at least 1 of the 12 forward searches for the 33-mRNA score.

### Definition of a nasal viral score

We calculated the nasal viral score for samples using the geometric mean of the normalized, log2-transformed expression of the up-regulated genes minus that of the down-regulated genes from the final gene signature. We scaled the scores for comparison between datasets. To measure the performance, we used the metrics of the receiver operating characteristic (ROC) curve and area under curve (AUROC) of the selected biomarkers.

### Pathway analysis

We performed Gene Set Enrichment Analysis using the *enrichGO* function in R package *clusterProfiler* [43]*.* To understand the biological relevance of the biomarkers discovered through multi-cohort analysis, we tested the significance of the over-representation of genes reflected in Gene Ontology (GO) annotation and adjusted the *p*-values from the test using the Benjamini–Hochberg method.

### scRNA-Seq analysis

We downloaded the scRNA-seq data for (1) GSE176269 [44] from the NCBI GEO and (2) SCP1289 from Single Cell Portal ([45] https://singlecell.broadinstitute.org/single_cell/study/SCP1289/impaired-local-intrinsic-immunity-to-sars-cov-2-infection-in-severe-covid-19). We performed quality control and processed both datasets separately with Seurat [46]. After normalizing read counts using ‘SCTransform,’ we performed principal component analysis (PCA), uniform manifold approximation and projection (UMAP), and shared nearest neighbors clustering on the data. Cell type annotation of clusters was performed with manual annotation using cell type markers.

### Logistic regression model using Inflammatix Machine Learning (IML) platform

To develop and train a logistic regression (LOGR) model, we used our in-house Inflammatix Machine Learning (IML) platform. This included only datasets with healthy controls. First, we co-normalized the samples across platforms using healthy control (HC) samples with a modified version of the ComBat empirical Bayes normalization method known as Combat CO-Normalization Using conTrols (COCONUT) (29). This approach makes one strong assumption: HC samples from different cohorts represent the same distribution. In short, HC samples from each platform undergo ComBat co-normalization without covariates. The co-normalized discovery data, comprising 6 training datasets a total of 382 samples, was used for training the LOGR model using IML. The training procedure comprised 1000 hyperparameter searches based on machine learning best practices. Furthermore, we used the 33 mRNAs post co-normalization to train the model. The locked model was then applied to the four validation datasets.

## Results

### Data collection, curation, and preprocessing

We identified 10 independent datasets composed of 1555 bulk transcriptome profiles from nasal samples of healthy controls (HC) or patients with viral ARIs or non-viral ARI (Table 1) [18, 21–23, 32, 34–36]. These 10 datasets enrolled infants, children, and adults with viral ARI or non-viral ARI across 5 countries (Table 1, Methods). Patients with non-viral ARI included those with bacterial pneumonia and non-infectious pulmonary conditions. Collectively, these datasets represented a broad spectrum of biological, clinical, and technical heterogeneity representative of the real-world patient population.

We chose 6 out of the 10 datasets as “discovery” datasets. The discovery datasets comprised 382 samples (236 viral ARI and 146 HCs). The remaining 4 datasets comprised 1173 samples (857 viral ARI, 251 non-viral ARI, and 65 HCs) were used for “validation” (Table 1). Our choice for using 6 datasets for discovery was based on three reasons. First, we have previously demonstrated that using 4–5 datasets comprising approximately 250–300 samples provides sufficient statistical power to detect differential expression even with higher between-dataset heterogeneity [26]. We had > 80% statistical power for detecting absolute effect size (ES) > 0.55 at a *p*-value of 0.05 even with high between-dataset heterogeneity (Additional file 1: Figure S1) [47]. Second, this division into discovery and validation datasets allowed us to use a larger number of samples with more clinical heterogeneity for validation while maintaining sufficient statistical power for discovery. Third, these 6 datasets represented broad biological (virus types and strains, age, sex), clinical (patient populations from 5 countries, severity), and technical heterogeneity (gene expression profiling platforms) to ensure the discovery of a robust gene signature (Table 1). However, the discovery datasets did not include any samples from patients with non-viral ARI. All datasets that included non-viral ARI patients were used as validation to ensure a more heterogeneous control population in the validation datasets. Using a higher number of samples with more clinical heterogeneity (i.e., patients with non-viral ARI) for validation provides strong evidence that our signature is not overfitted and is robust to the unseen clinical heterogeneity of the real-world patient population.

### 119 genes are consistently differentially expressed in viral ARIs in nasal swab samples

**Fig. 1:**
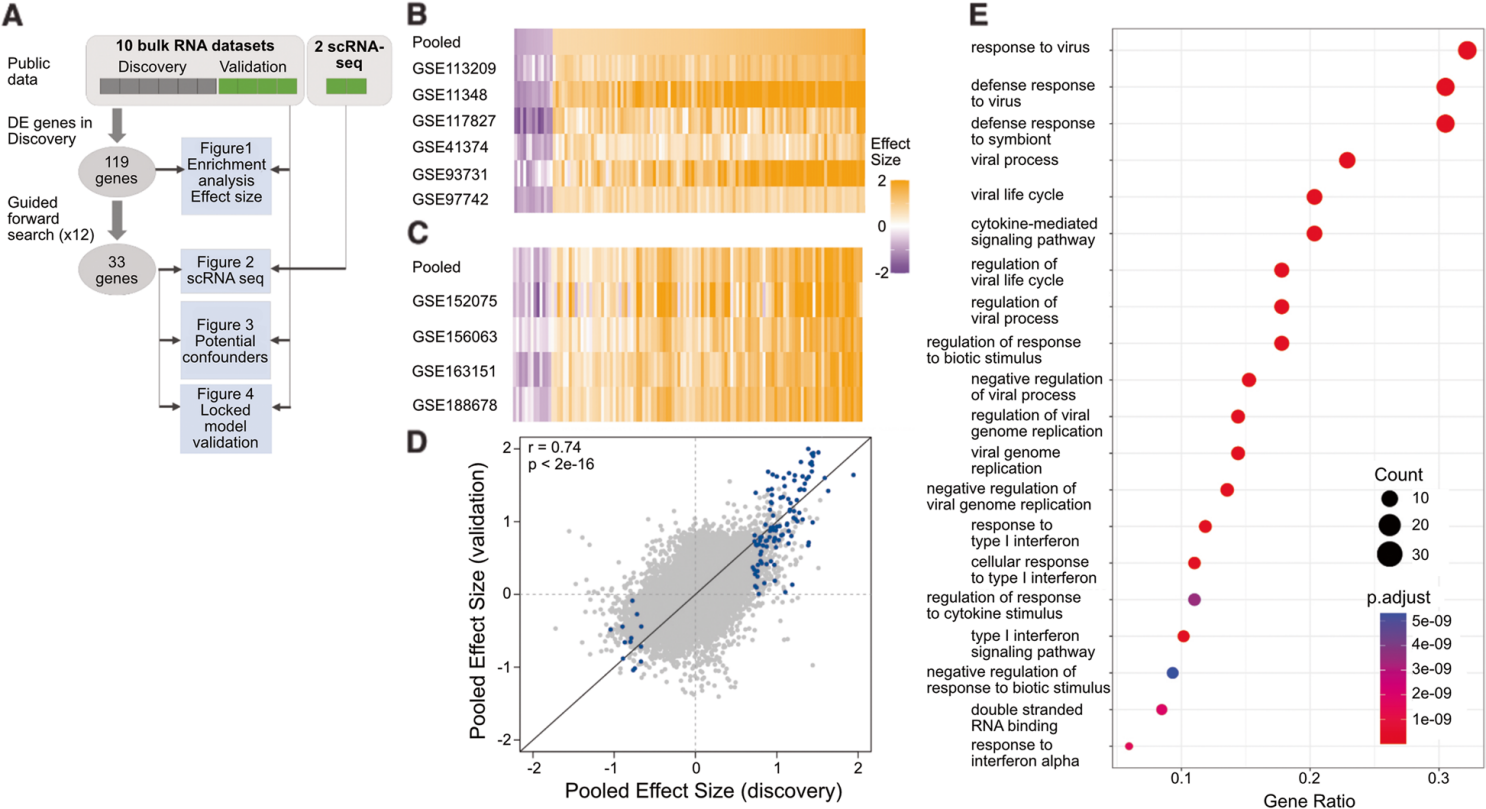
Multicohort analysis identifies 328 differentially expressed genes in viral ARI nasal samples. **A** Graphical representation of the analysis. **B**,** C** Effect size heatmap of 119 differentially expressed genes in discovery (**B**) and validation (**C**).** D** Scatter plot showing the effect size correlation between discovery and validation datasets for all genes (grey) and 119 differentially expressed genes measured in all studies (blue).** E** Ontology enrichment analysis of 119 genes

Multi-cohort analysis using MetaIntegrator identified 328 genes differentially expressed (|ES|≥ 0.6, FDR ≤ 10%) in nasal swabs of patients with viral ARIs compared to HCs using the 6 discovery datasets (Additional File 2: Table S1). Out of these 328 genes, 119 genes were measured across all datasets and were differentially expressed in the same direction in the discovery and validation cohorts (Fig. 1B–C) with highly correlated effect sizes between training and validation datasets (*r* = 0.74, *p* < 2e − 16; Fig. 1D). As expected, gene enrichment analysis found that these genes are involved in pathways associated with the host response to viral infection including defense response to viruses, regulation of innate immune response, cytokine-mediated response, and response to type 1 interferon (Fig. 1E).

### A 33-mRNA immune response signature distinguishes viral ARI from healthy controls

We have repeatedly demonstrated that gene signatures identified using a multi-cohort analysis framework could be further reduced to a subset of genes that is more amenable to translation to a point-of-care clinical test (27,31,40–43). While we note that many sub-combinations of the differentially expressed genes could be optimized for diagnosis, we have previously described a greedy forward search [15] to iteratively (“Methods”) identify a smaller number of genes that can be translated into a point-of-care test. Our greedy forward search identified a 33-mRNA signature (24 over-expressed, 9 under-expressed) that was consistently differentially expressed in patients with viral ARI from HCs in the discovery datasets and from HCs and patients with non-viral ARI in validation datasets (“Methods”; Table S1 and Additional file 1: Figure S2).

### Myeloid cells are the primary source of the 33-mRNA signature

We used single-cell RNA sequencing (scRNA-seq) profiles of 43,814 cells from nasal samples of 78 individuals (55 viral infections, 23 HCs) reported in two independent cohorts (SCP1289 [48] and GSE176269 [44]) to identify the cell types that express the 33 genes. Specifically, GSE176269 profiled 18,913 cells from nasal wash samples from 20 individuals (6 SARS-COV-2, 8 influenza A, and 6 HCs). The other dataset, SCP1289, profiled 24,901 cells from nasopharyngeal samples from 58 individuals (35 SARS-CoV-2 patients, 6 respiratory failure patients, and 17 HCs). Because of the differences in sample types (nasopharyngeal vs nasal wash), we did not integrate both studies into a single dataset.

**Fig. 2:**
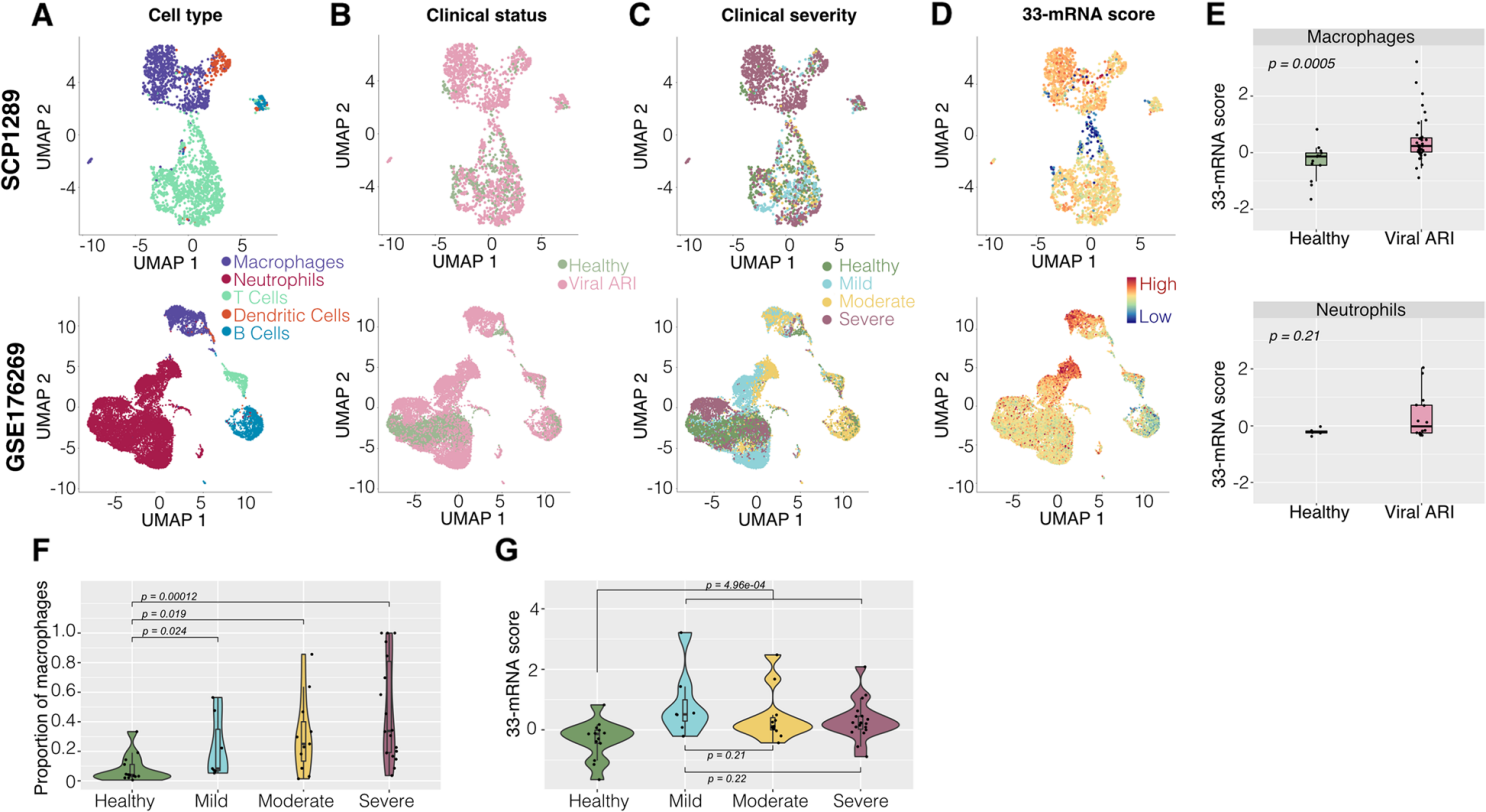
33-mRNA score reflects transcriptional changes in macrophages. UMAP representation of scRNA-seq data from SCP1289 and GSE176269. **A–D** represent clustering based on cell type (**A**), clinical status (**B**), severity (**C**), and 33-mRNA score (**D**). High score is attributable to myeloid cells (macrophages, dendritic, and neutrophil cells). Color coding represented in the upper panel applies to the lower panel as well. **E** Boxplots showing 33-mRNA score distribution in macrophages and neutrophils by clinical status. *p*-values were calculated using Wilcox test. **E** shares color code with **B**. **F** Proportion of macrophages out of immune cells sequences per sample as function of disease severity. **G** Distribution of pseudo-bulk 33-mRNA score in macrophages as function of disease severity. **F** and **G** share color code with **C**

Visualization using UMAP showed that the largest variation was by cell type (Fig. 2A) followed by the infection status (Fig. 2B) and severity (Fig. 2C). We defined the 33-mRNA signature score of a cell as the difference between the geometric mean of 24 over-expressed genes and that of 9 under-expressed genes. Macrophages and neutrophils had the highest scores (Fig. 2D) and pseudo-bulk analysis (“Methods”) showed that the 33-mRNA scores were significantly higher in macrophages from patients with viral ARI (Fig. 2E), but not in the other cell types. These results are in line with the conserved host response to viral infection in blood [17] and provide further evidence that myeloid cells are also the primary source of the conserved host response at the site of infection. Importantly, we found that proportions of macrophages increased with the severity of viral ARI (Fig. 2F), but there was no association between the 33-mRNA score at the single-cell level and the severity of viral ARI (Fig. 2G), further suggesting that increased 33-mRNA score is due to the change in the proportion of macrophages in the respiratory tract.

### The 33-mRNA score distinguishes patients with viral ARI from those with non-viral ARI and healthy controls

**Fig. 3:**
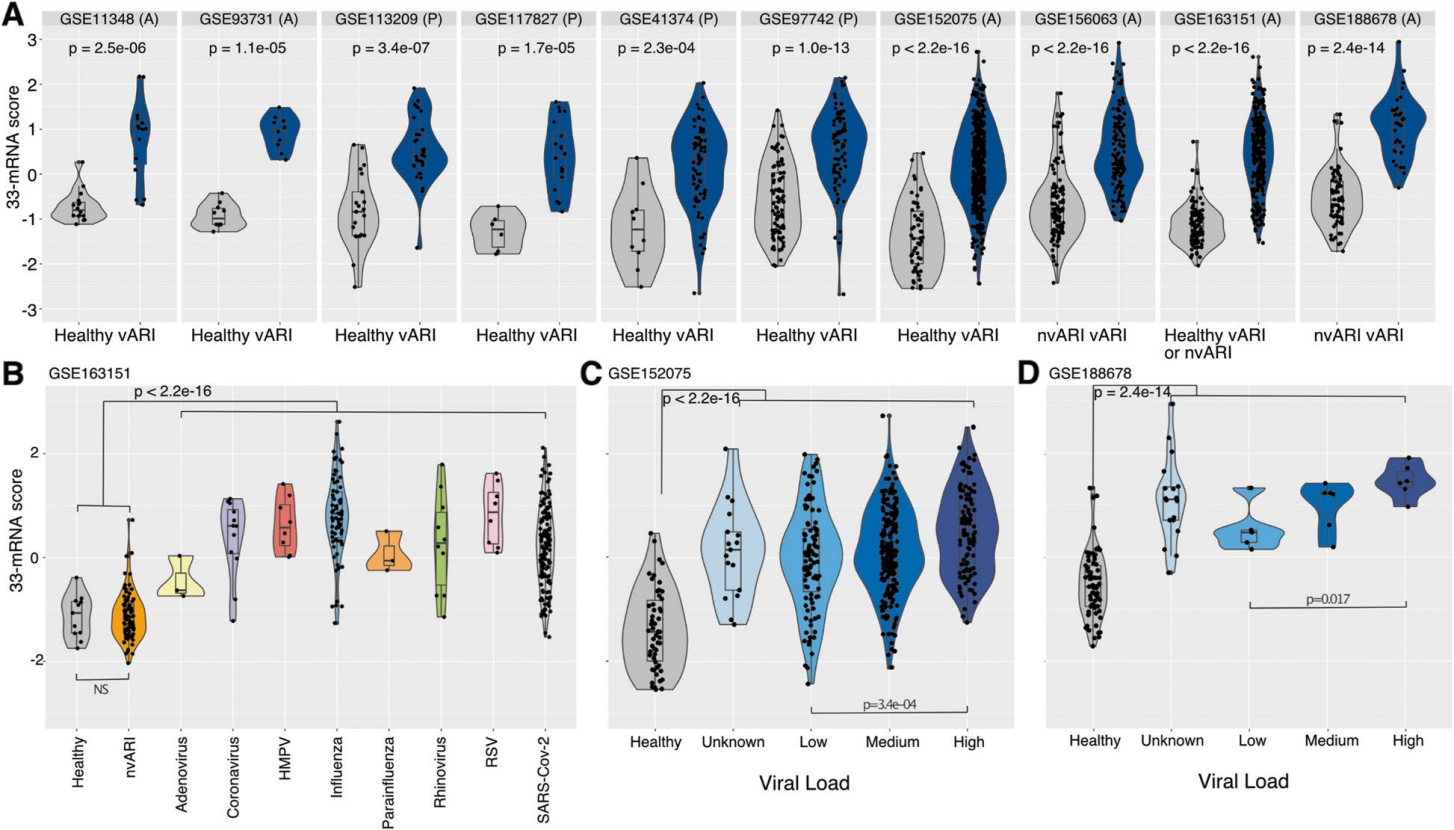
33-mRNA signature is robust to real world heterogeneity. Distributions of sample-specific 33-mRNA scores across multiple, real life, potential confounding: age (**A**), virus type (**B**), and viral load (**C**, **D**). In all panels, y-axis is 33-mRNA score.** A** Control groups (either healthy, HC or non-viral ARI, nvARI) are grey while viral ARI (vARI) is blue. *p*-value was calculated using Wilcox test.** B** Distribution of 33-mRNA score across different viruses in GSE163151. **C**, **D** Distribution of 33-mRNA score across different viral loads in GSE152075 and GSE188678, respectively. Viral load was defined by cycle threshold (Ct) of N1 target region of SARS-CoV-2 virus (**C**) or by RPM of SARS-CoV-2 virus (**D**). **C** and **D** share Y axis with **B**

Similar to the 33-mRNA score for a single cell, we defined a sample-level 33-mRNA score as the difference between the geometric mean of the overexpressed genes and that of the under-expressed genes in all cells attributed to a particular sample. As expected, sample-level 33-mRNA scores for patients with viral ARI were significantly higher than those for HCs in the discovery datasets for pediatric and adult patients (*p* < 3e − 04; Fig. 3A). More importantly, three validation datasets included patients with non-viral ARI. In these datasets, the 33-mRNA scores for patients with viral ARI were significantly higher than those for HCs and patients with non-viral ARI (*p* < 3e − 14; Fig. 3A–B), although samples from non-viral ARI patients were not used to identify the 33-mRNA signature. In one study, GSE163151, where both healthy and non-viral ARI samples were present, 33-mRNA scores did not differ between the two control groups (Fig. 3B). These results further underscore the generalizability and specificity of the 33-mRNA to distinguish patients with viral ARI from HCs or patients with non-viral ARI.

### The 33-mRNA score is not confounded by age or viral type and is correlated with viral load

Multiple studies have described differences in immune response in children and adults [35, 49]. Therefore, we investigated whether age has any impact on the 33-mRNA signature. As expected, the 33-mRNA score was significantly higher in children and adults with viral ARI compared to healthy controls in the discovery datasets. However, there was no difference in the 33-mRNA scores between children and adults with viral ARI (Fig. 3A). Furthermore, linear regression model using viral ARI, age, and sex as independent variables found that only infection status was significantly associated with the 33-mRNA score (*p* < 2e − 16), whereas age and sex were not associated with 33-mRNA scores (*p* > 0.11). We also examined the effects of sex and age by study and found no significant correlation between 33-mRNA score and either sex (Additional file 1: Figure S3) or age (Additional file 1: Figure S4) in any of the 8 studies where age and sex information was available. However, we note that > 85% of the samples in the discovery datasets were from infants and children, whereas the validation datasets were comprised exclusively of adults (Table 1). Hence, although our results strongly suggest that our multi-cohort analysis identified the host response to viral infections in the nasal mucosa that is conserved across different age groups, it should be further validated in pediatric cohorts.

Next, we investigated whether the 33-mRNA signature is impacted by the type or load of viral infection. GSE163151 included samples from multiple viral infections whereas GSE152075 and GSE188678 included samples with a range of viral loads. We did not observe a significant effect of the type of virus on the 33-mRNA score across all 8 types of viruses contained in GSE163151 (Fig. 3B). Notably, the 33-mRNA score was significantly higher in patients with a high viral load than those with a low viral load in GSE152075 (*p* = 3e − 04) and GSE188678 (*p* = 0.017) (Fig. 3C–D). These results suggest that 33-mRNA is not affected by a type of virus and correlated with viral load in the respiratory tract. Together, our analysis strongly suggests that 33-mRNA is likely to be generalizable in clinical settings regardless of the viral type and loads.

### The locked 33-mRNA logistic regression-based classifier generalizes to new datasets with high accuracy

**Fig. 4:**
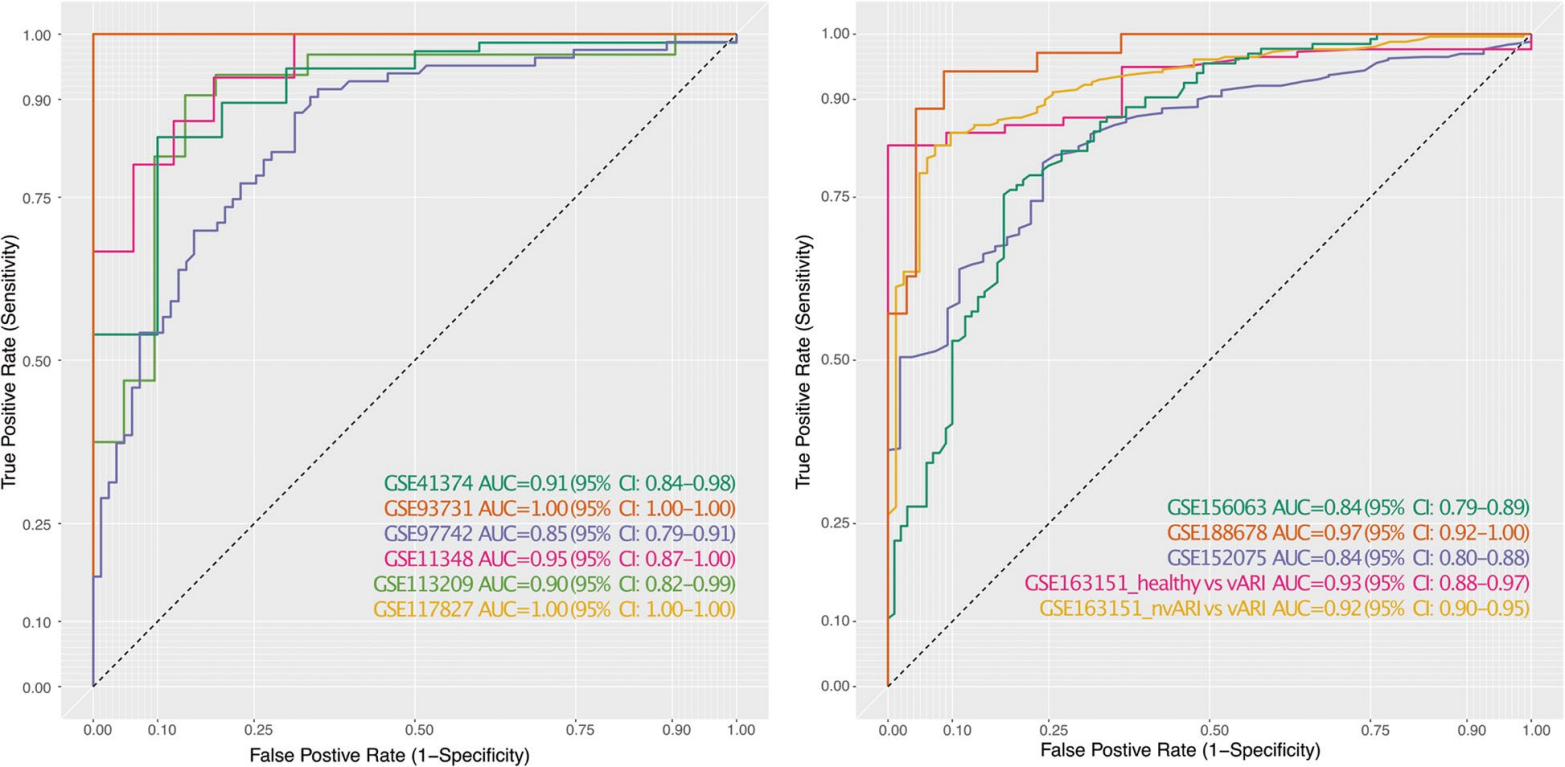
Performance of LOGR model of the 33 mRNAs in training and validation datasets. Datasets were split the same way as in original discovery (Table 1). Logistic Regression model was trained using expression values of the 33 mRNAs in 6 discovery datasets (**A**) achieving summary AUC = 0.94 and then applied as a locked model to 4 validation datasets without co-normalization (**B**) achieving summary AUC of 0.89. In validation, GSE163151 was split into two discovery datasets to represent healthy and non-viral ARI separately

Clinical translation of a diagnostic signature requires a locked classification model that can be applied to new datasets without the need for retraining or fine-tuning. The generalizability of the 33-mRNA signature across datasets despite the presence of biological, clinical, and technical heterogeneity strongly suggests that it is a reasonable candidate for creating such a classifier. We used our previously described Inflammatix Machine Learning (IML) platform [31] to develop a logistic regression (LOGR)-based classifier using the discovery datasets, which classified patients with viral ARI from HCs with summary AUROC of 0.94 (range: 0.85–1.00; Fig. 4A). Next, we applied this LOGR classifier “as is” (i.e., locked without any modifications) to the four validation datasets previously unseen to the model. We note that the validation datasets were not co-normalized with the discovery datasets or with each other. The locked 33-mRNA LOGR classifier had a summary AUROC of 0.89 (range: 0.84–0.97) in the four validation datasets (Fig. 4B). At the optimal Youden threshold, the locked LOGR classifier had 82.87% sensitivity and 82.4% specificity for the diagnosis of viral ARI in the validation datasets. Taken together, these results highlight the robustness and generalizability of the 33-mRNA signature for future diagnostic development.

## Discussion

To our knowledge, this is the first multi-cohort analysis of host response in nasal swab samples across viral ARIs. The goal of this study was to evaluate the potential for clinical development of a host response gene expression signature that identifies viral ARIs in real-world clinical settings. To this end, we applied a multicohort analysis approach to 1555 samples from 10 publicly available datasets, dividing them into discovery and validation groups. We identified a 33-mRNA signature in nasal samples that distinguished viral ARI with AUC > 0.9 in both discovery and validation datasets. We showed that the 33-mRNA signature generalizes to multiple viruses and is robust to biological heterogeneity such as viral type or load in the real-world patient population.

A diagnostic test for viral ARIs must be robust to clinical heterogeneity. Specifically, it should be able to differentiate patients with viral ARIs from those who present with similar symptoms but have non-viral ARIs to generalize to real-world settings where viral types and loads are highly variable. Using a large number of samples (427 viral ARI, 251 non-viral ARI) across three independent validation cohorts, we showed that although we identified the 33 mRNAs using HCs, the model also distinguished patients with viral ARI from those with non-viral ARI with > 80% sensitivity and specificity. Importantly, one of the validation datasets, GSE156063, included non-viral ARI samples from patients with bacterial pneumonia as well as non-infectious lung and airway conditions but did not provide patient-level information. In GSE156063, the 33-mRNA score had an AUROC of 0.84, which further demonstrates its robustness to clinical heterogeneity in diagnosing viral ARIs. In addition, three datasets (GSE163151, GSE152075, and GSE188678) provided information about virus type and viral load. The 33-mRNA model had high accuracy for diagnosis regardless of virus type (AUC = 0.92) and viral load (AUCs = 0.97 and 0.84).

Accurate and timely determination of broad sources of infection in ARI patients would expedite proper care, minimize unnecessary use of antibiotics, and ultimately save lives. It would also provide a path forward for monitoring clinically relevant emerging viruses. Such a test, in conjunction with negative results from viral screening panels and clinical presentation, would identify the samples that are likely to harbor unidentified viruses posing a health risk and thus warranting further interrogation. In fact, given the recent technological advances, it would be possible to integrate the 33-mRNA signature with viral screening panels as a single diagnostic test. Such integration would also allow confidently ruling out viral infection in patients who tested negative for a panel of respiratory viruses and do not show the presence of host response to viral infections.

Cytokine-mediated response and inflammation play a significant role in defense against a viral infection [50, 51]. For example, Yu et al. have noted a strong type I interferon response in the nasal transcriptome of patients with viral ARI [22]. Studies by Landry et al. and Cheemarla et al. also demonstrated upregulation of interferon induced cytokines in nasopharyngeal samples of patients with viral infection, both at mRNA and protein levels. Consistent with previous studies, our pathways enrichment analysis of the 119 genes highlights defense response to viruses, regulation of innate immunity, and response to type I interferon [19, 25]. The most upregulated genes in our metanalysis, such as ISG15, various IFI genes, and RSAD2 (Table S1) are also all known interferon-induced genes. Consistently, many of the selected 33 mRNAs are also interferon-induced. Examination of scRNA-seq studies provides further evidence that the 33-mRNA signature reflects changes in both myeloid cell expression and composition, particularly an increase in macrophage fractions as well as cellular changes in both macrophage and neutrophil expression. Indeed, *CD163*, a monocyte and macrophage-specific gene, is also part of the 33-mRNA signature. Collectively, these results are in line with previous studies that conserved host response to viral infection is dominated by myeloid cells [17].

Previous studies have suggested that epithelial cells make a major contribution to the early host response to viral infection [22, 48]. For example, Ziegler et al. described a very strong epithelial cell response, with myeloid cells playing a relatively larger role in very severe COVID. However, our 33-mRNA signature is preferentially expressed in myeloid cells. This discrepancy raises the question of whether the 33-mRNA signature might be biased against the epithelial cell response, and if so, would this cause it to lose sensitivity for early infections and/or infections of mild-moderate severity. We note that the 33-mRNA signature was derived from datasets that included the entire gamut of cells present in the nasal wash and swab samples, including epithelial cells. Because the multicohort analysis framework, MetaIntegrator, used in our analysis prioritizes consistency of expression despite the biological, clinical, and technical heterogeneity across datasets, it is very likely that the 33-mRNA signature emerged due to the robustness of host response in myeloid cells, whereas the sampling biases across cohorts may have affected the robustness of expression from epithelial cells. This is an important consideration for clinical translation as we would like to minimize variability due to sampling bias at point-of-care. If there was consistent expression in epithelial cells across all datasets, our framework would have prioritized it. In other words, our discovery process is unlikely to be intrinsically biased against selecting the genes expressed in epithelial cells.

Our study has a few limitations. First, COCONUT co-normalization of training datasets required to build the regression model may have obfuscated some biological signals. However, our validation datasets were not co-normalized, which in turn demonstrated that the effects of acute viral infection on the host immune response are robustly detectable across cohorts. Second, we validated the 33-mRNA signature in retrospective cohorts that used only microbiologically confirmed patients in a case–control design. The performance characteristics of the 33-mRNA signature will differ in patient cohorts that rely on non-microbiological adjudication methods, which are known to suffer from high inter-rater variations, leading to a variable standard. The 33-mRNA signature should be validated in additional prospective cohorts from other populations and where the microbiological diagnosis is not available. In such a study, RNAseq could be used to validate viral presence and load in an unbiased manner. Third, critical considerations for clinical use are the temporal and clinical resolution of the test. None of the studies included in our analysis profiled patients longitudinally, provided time since symptom onset, or documented severity. Given the widespread acceptance of home nasal swab tests, there is high likelihood that patients will present at different time since symptom onset, this data should be included in future studies. Hence, future studies would need to include individuals with different, documented levels of infection severity as well as different times since symptom onsets to assess diagnostic performance of the signature. Fourth, our validation cohorts, which included patients with non-viral ARI, did not provide sample-level information. Therefore, we could not further explore accuracy of our classifier in distinguishing viral ARI from specific non-viral ARIs. Fifth, although we found no differences in the 33-mRNA scores between adult and pediatric patients with viral ARI in the discovery datasets, all validation datasets, which also included all non-viral ARI samples, only included adult patients. Hence, further validation in pediatric cohorts is required to assess generalizability of the classifier across the entire age spectrum, and in pediatric patients with non-viral ARI. Future studies would focus on testing our signature and the classifier in pediatric patients and in elderly patients to assess the impact of aging. Sixth, none of the studies included in our analysis provided information on comorbidities. Hence, we could not assess how our 33-mRNA signature would perform in patients with comorbidities, especially immune system-related disorders. Future studies validating our 33-mRNA signatures should focus on addressing this limitation.

Despite these limitations, we have successfully utilized multicohort analyses of heterogeneous data to repeatedly identify robust host response signatures that diagnose the presence of an infection [15], differentiate bacterial vs viral infection [29, 52], predict severity of infection [17, 53–56], and in some cases identify the infecting pathogen [14, 16] in blood samples. We and others have validated these signatures in independent prospective cohorts [57, 58]. Combined with machine learning-based classifiers, we have moved several of these signatures to clinical development and demonstrated their accuracy in multiple prospective cohorts [30, 31, 56, 59, 60]. Our long-term goal is to extend the similar capability to nasal swab samples, and the current work is the first step in that process that demonstrates the feasibility of our approach.

## Conclusions

Our study of host response profiles in the nasal samples of viral ARI patients identified a robust 33-mRNA signature diagnostic of viral ARI that is primarily driven by myeloid cells and conserved across different age groups. This signature forms a foundation for the development of ‘RespVerity’—a diagnostic test that will identify viral ARIs using nasal swab samples. Furthermore, such a test could integrate pathogen detection as well as host response on a single diagnostic platform using nasal swab samples. This test will allow rapid confirmation of viral infection when a pathogen cannot be identified and thereby reduce the misuse of antibiotics and facilitate pathogen surveillance.

### Supplementary Information

**Additional file 1: Figure S1:** Power analysis.** Figure S2:** Effect Size heatmap of 33-mRNA signature in discovery and validation datasets. **Figure S3:** Distributions of 33-mRNA score by sex in 8 studies. **Figure S4:** Distributions 33-mRNA score by sample age in 8 studies.

**Additional file 2: Table S1.** List of differentially expressed genes.

## Data Availability

The datasets supporting the conclusions of this study are publicly available from GEO. Deidentified transcriptomic and phenotype data for the analyzed datasets are available at: GSE113209 https://www.ncbi.nlm.nih.gov/geo/query/acc.cgi?acc=GSE113209 [32] GSE11348 https://www.ncbi.nlm.nih.gov/geo/query/acc.cgi?acc=GSE11348 [21] GSE117827 https://www.ncbi.nlm.nih.gov/geo/query/acc.cgi?acc=GSE117827 [22] GSE41374 https://www.ncbi.nlm.nih.gov/geo/query/acc.cgi?acc=GSE41374 [33] GSE93731 https://www.ncbi.nlm.nih.gov/geo/query/acc.cgi?acc=GSE93731 [34] GSE97742 https://www.ncbi.nlm.nih.gov/geo/query/acc.cgi?acc=GSE97742 [23] GSE152075 https://www.ncbi.nlm.nih.gov/geo/query/acc.cgi?acc=GSE152075 [35] GSE156063 https://www.ncbi.nlm.nih.gov/geo/query/acc.cgi?acc=GSE156063 [18] GSE163151 https://www.ncbi.nlm.nih.gov/geo/query/acc.cgi?acc=GSE163151 [36] GSE188678 https://www.ncbi.nlm.nih.gov/geo/query/acc.cgi?acc=GSE188678 [37] GSE176269 https://www.ncbi.nlm.nih.gov/geo/query/acc.cgi?acc=SE176269 [44] MetaIntegrator package is available from https://CRAN.R-project.org/package=MetaIntegrator. IML software includes proprietary portions and is not available; however, LOGR models, used to create the final classifier, can be reproduced with open source tools in either R or Python.
