# Supplementary material for: A machine learning classifier using 33 host immune response mRNAs accurately distinguishes viral and non-viral acute respiratory illnesses in nasal swab samples
Source: Genome Med. 2023 Aug 28;15:64. doi: 10.1186/s13073-023-01216-0 (PMC10463681; doi:10.1186/s13073-023-01216-0)
Supplement: Supplementary file 1 — Additional file 1: Figure S1: Power analysis. Figure S2: Effect Size heatmap of 33-mRNA signature in discovery and validation datasets. Figure S3: Distributions of 33-mRNA score by sex in 8 studies. Figure S4: Distributions 33-mRNA score by sample age in 8 studies. [file 13073_2023_1216_MOESM1_ESM.docx]

**SUPPLEMENTARY FIGURES**

**A machine learning classifier using 33 host immune response mRNAs accurately distinguishes viral and non-viral acute respiratory illnesses in nasal swab samples**

Rushika Pandaya^1#^, Yudong D. He^1#^, Timothy E. Sweeney^1^, Yehudit Hasin-Brumshtein^1±^, Purvesh Khatri^1±*^

^1^Inflammatix Inc., Sunnyvale, CA 94085, USA

# These authors are no longer with Inflammatix at time of submission

^±^ Co-senior authors

^*^ Correspondence: Purvesh Khatri (pkhatri@stanford.edu)

**Figure S1:** Power analysis indicated >80% statistical power for detecting absolute effect size (ES) > 0.55 at p-value of 0.05 even with high between-dataset heterogeneity

**Figure S2:** Effect Size heatmap of 33-mRNA signature in discovery and validation datasets

**Figure S3:** Distributions of 33-mRNA score by sample sex for 8 studies that provided sex information. P-value for mean comparison is indicated.

Control

vARI

B

A

C

**Figure S4:** 33-mRNA score by sample age for 8 studies that provided age information. The 33-mRNA score is on y axis. A. Violin plots of score by age group (<1 year old, 1-5, 6-18, 19-40, 41-60, 60-100). B and C show a scatter plot of all samples age and 33-mRNA score. Lines show linear regression fit with 95% confidence interval and R and p values per each study. Colors and point shape indicate study as per legend between B and C.
