# Supplementary material for: A machine learning classifier using 33 host immune response mRNAs accurately distinguishes viral and non-viral acute respiratory illnesses in nasal swab samples
Source: Genome Med. 2023 Aug 28;15:64. doi: 10.1186/s13073-023-01216-0 (PMC10463681; doi:10.1186/s13073-023-01216-0)
Supplement: Supplementary file 2 — Additional file 2: Table S1. List of differentially expressed genes. [file 13073_2023_1216_MOESM2_ESM.docx]

**Supplementary Table 1. List of differentially expressed genes.**

| SYMBOL | ENTREZID | pooledES | change | NStudies | In_119_genes | 33mRNA model |
| --- | --- | --- | --- | --- | --- | --- |
| GNLY | 10578 | 0.911 | + | 10 | 1 | 1 |
| CD163 | 9332 | 1.318 | + | 10 | 1 | 1 |
| GLRX | 2745 | 1.393 | + | 10 | 1 | 1 |
| STXBP2 | 6813 | 0.778 | + | 10 | 1 | 1 |
| NCOA7 | 135112 | 0.808 | + | 10 | 1 | 1 |
| C1QB | 713 | 1.073 | + | 10 | 1 | 1 |
| SLC4A2 | 6522 | 0.75 | + | 10 | 1 | 1 |
| IFITM1 | 8519 | 1.589 | + | 10 | 1 | 1 |
| TMSB10 | 9168 | 1.19 | + | 10 | 1 | 1 |
| TIMP1 | 7076 | 1.104 | + | 10 | 1 | 1 |
| ATF4 | 468 | 0.735 | + | 10 | 1 | 1 |
| IFITM3 | 10410 | 1.632 | + | 10 | 1 | 1 |
| LY6E | 4061 | 1.386 | + | 10 | 1 | 1 |
| IFI6 | 2537 | 1.447 | + | 10 | 1 | 1 |
| OASL | 8638 | 1.43 | + | 10 | 1 | 1 |
| EPSTI1 | 94240 | 1.43 | + | 10 | 1 | 1 |
| IFIT1 | 3434 | 1.449 | + | 10 | 1 | 1 |
| XAF1 | 54739 | 1.428 | + | 10 | 1 | 1 |
| ISG15 | 9636 | 1.943 | + | 10 | 1 | 1 |
| IFIT2 | 3433 | 1.434 | + | 10 | 1 | 1 |
| C1GALT1 | 56913 | 0.732 | + | 10 | 1 | 1 |
| IFI44L | 10964 | 1.555 | + | 10 | 1 | 1 |
| RSAD2 | 91543 | 1.511 | + | 10 | 1 | 1 |
| SIGLEC1 | 6614 | 1.44 | + | 10 | 1 | 1 |
| EIF3L | 51386 | -0.891 | - | 10 | 1 | 1 |
| IK | 3550 | -0.775 | - | 10 | 1 | 1 |
| RPL3 | 6122 | -0.765 | - | 10 | 1 | 1 |
| PRPF8 | 10594 | -0.8 | - | 10 | 1 | 1 |
| PPP1R14C | 81706 | -1.044 | - | 10 | 1 | 1 |
| KIF3B | 9371 | -0.719 | - | 10 | 1 | 1 |
| TPRG1L | 127262 | -0.79 | - | 10 | 1 | 1 |
| TOMM34 | 10953 | -0.897 | - | 10 | 1 | 1 |
| HLF | 3131 | -0.666 | - | 10 | 1 | 1 |
| TRIM38 | 10475 | 1.036 | + | 10 | 1 | 0 |
| MS4A7 | 58475 | 0.865 | + | 10 | 1 | 0 |
| FKBP5 | 2289 | 0.816 | + | 10 | 1 | 0 |
| C1QC | 714 | 0.977 | + | 10 | 1 | 0 |
| SLC16A3 | 9123 | 0.848 | + | 10 | 1 | 0 |
| CYBB | 1536 | 0.727 | + | 10 | 1 | 0 |
| GIMAP4 | 55303 | 0.914 | + | 10 | 1 | 0 |
| ITGA5 | 3678 | 0.705 | + | 10 | 1 | 0 |
| NAGK | 55577 | 0.763 | + | 10 | 1 | 0 |
| OAS1 | 4938 | 0.94 | + | 10 | 1 | 0 |
| NCKAP1L | 3071 | 0.742 | + | 10 | 1 | 0 |
| FCGR1A | 2209 | 0.99 | + | 10 | 1 | 0 |
| CTSL | 1514 | 0.969 | + | 10 | 1 | 0 |
| TAP1 | 6890 | 1.128 | + | 10 | 1 | 0 |
| SAMD9 | 54809 | 1.097 | + | 10 | 1 | 0 |
| NADK | 65220 | 0.874 | + | 10 | 1 | 0 |
| TYMP | 1890 | 0.96 | + | 10 | 1 | 0 |
| SHISA5 | 51246 | 0.928 | + | 10 | 1 | 0 |
| NT5C3A | 51251 | 1.125 | + | 10 | 1 | 0 |
| FPR3 | 2359 | 0.954 | + | 10 | 1 | 0 |
| SIGLEC10 | 89790 | 0.768 | + | 10 | 1 | 0 |
| GCH1 | 2643 | 0.879 | + | 10 | 1 | 0 |
| FCN1 | 2219 | 0.876 | + | 10 | 1 | 0 |
| IL10RA | 3587 | 0.814 | + | 10 | 1 | 0 |
| GRN | 2896 | 0.977 | + | 10 | 1 | 0 |
| FCER1G | 2207 | 1.179 | + | 10 | 1 | 0 |
| IFITM2 | 10581 | 1.024 | + | 10 | 1 | 0 |
| OAS3 | 4940 | 1.296 | + | 10 | 1 | 0 |
| CARD16 | 114769 | 0.78 | + | 10 | 1 | 0 |
| HERC5 | 51191 | 1.278 | + | 10 | 1 | 0 |
| IRF7 | 3665 | 1.212 | + | 10 | 1 | 0 |
| LGALS9 | 3965 | 1.057 | + | 10 | 1 | 0 |
| C3AR1 | 719 | 0.971 | + | 10 | 1 | 0 |
| SP100 | 6672 | 0.741 | + | 10 | 1 | 0 |
| ZBP1 | 81030 | 1.276 | + | 10 | 1 | 0 |
| FRMD3 | 257019 | 0.799 | + | 10 | 1 | 0 |
| HELZ2 | 85441 | 1.207 | + | 10 | 1 | 0 |
| DDX58 | 23586 | 1.222 | + | 10 | 1 | 0 |
| ZC3HAV1 | 56829 | 0.966 | + | 10 | 1 | 0 |
| KLF6 | 1316 | 0.762 | + | 10 | 1 | 0 |
| IFIT3 | 3437 | 1.386 | + | 10 | 1 | 0 |
| LILRB4 | 11006 | 0.728 | + | 10 | 1 | 0 |
| OAS2 | 4939 | 1.411 | + | 10 | 1 | 0 |
| DDX60 | 55601 | 1.15 | + | 10 | 1 | 0 |
| SERPING1 | 710 | 1.294 | + | 10 | 1 | 0 |
| CST7 | 8530 | 0.8 | + | 10 | 1 | 0 |
| IFIT5 | 24138 | 1.246 | + | 10 | 1 | 0 |
| CD69 | 969 | 0.83 | + | 10 | 1 | 0 |
| SAMD9L | 219285 | 1.344 | + | 10 | 1 | 0 |
| CMPK2 | 129607 | 1.392 | + | 10 | 1 | 0 |
| TNFSF13B | 10673 | 0.954 | + | 10 | 1 | 0 |
| DDX60L | 91351 | 0.894 | + | 10 | 1 | 0 |
| ZFYVE26 | 23503 | 0.915 | + | 10 | 1 | 0 |
| IFIH1 | 64135 | 1.169 | + | 10 | 1 | 0 |
| ISG20 | 3669 | 1.252 | + | 10 | 1 | 0 |
| DTX3L | 151636 | 0.938 | + | 10 | 1 | 0 |
| MX2 | 4600 | 1.086 | + | 10 | 1 | 0 |
| TNFSF10 | 8743 | 1.146 | + | 10 | 1 | 0 |
| SECTM1 | 6398 | 1.144 | + | 10 | 1 | 0 |
| CCR1 | 1230 | 1.004 | + | 10 | 1 | 0 |
| SP110 | 3431 | 1.141 | + | 10 | 1 | 0 |
| BATF | 10538 | 0.722 | + | 10 | 1 | 0 |
| CASP5 | 838 | 0.793 | + | 10 | 1 | 0 |
| STAT2 | 6773 | 1.161 | + | 10 | 1 | 0 |
| CXCL10 | 3627 | 1.338 | + | 10 | 1 | 0 |
| GBP1 | 2633 | 1.001 | + | 10 | 1 | 0 |
| MX1 | 4599 | 1.317 | + | 10 | 1 | 0 |
| PARP12 | 64761 | 1.157 | + | 10 | 1 | 0 |
| HERC6 | 55008 | 0.938 | + | 10 | 1 | 0 |
| TFEC | 22797 | 0.926 | + | 10 | 1 | 0 |
| PARP9 | 83666 | 0.893 | + | 10 | 1 | 0 |
| IFI44 | 10561 | 1.367 | + | 10 | 1 | 0 |
| TRIM22 | 10346 | 0.949 | + | 10 | 1 | 0 |
| CD48 | 962 | 0.795 | + | 10 | 1 | 0 |
| USP18 | 11274 | 1.216 | + | 10 | 1 | 0 |
| PARP14 | 54625 | 1.075 | + | 10 | 1 | 0 |
| APOL6 | 80830 | 0.928 | + | 10 | 1 | 0 |
| SLAMF7 | 57823 | 1.229 | + | 10 | 1 | 0 |
| GBP3 | 2635 | 0.752 | + | 10 | 1 | 0 |
| EIF2AK2 | 5610 | 1.045 | + | 10 | 1 | 0 |
| PIK3AP1 | 118788 | 0.86 | + | 10 | 1 | 0 |
| CASP1 | 834 | 0.989 | + | 10 | 1 | 0 |
| ALDH3A1 | 218 | -0.671 | - | 10 | 1 | 0 |
| PARVA | 55742 | -0.67 | - | 10 | 1 | 0 |
| PRDX5 | 25824 | -0.746 | - | 10 | 1 | 0 |
| GSTA1 | 2938 | -0.869 | - | 10 | 1 | 0 |
| HAVCR2 | 84868 | 1.218 | + | 9 | 0 | 0 |
| MS4A6A | 64231 | 1.084 | + | 9 | 0 | 0 |
| TLNRD1 | 59274 | 1.06 | + | 7 | 0 | 0 |
| LHFPL2 | 10184 | 1.166 | + | 9 | 0 | 0 |
| MSR1 | 4481 | 0.694 | + | 9 | 0 | 0 |
| TPP1 | 1200 | 0.993 | + | 9 | 0 | 0 |
| ITPRIPL2 | 162073 | 0.698 | + | 9 | 0 | 0 |
| GIMAP1 | 170575 | 0.926 | + | 7 | 0 | 0 |
| ITGB2 | 3689 | 0.739 | + | 9 | 0 | 0 |
| C1orf162 | 128346 | 1.083 | + | 8 | 0 | 0 |
| FAM20A | 54757 | 0.851 | + | 8 | 0 | 0 |
| FZD2 | 2535 | 1.041 | + | 6 | 0 | 0 |
| SLC39A8 | 64116 | 0.666 | + | 9 | 0 | 0 |
| GPBAR1 | 151306 | 0.785 | + | 7 | 0 | 0 |
| ENG | 2022 | 0.935 | + | 8 | 0 | 0 |
| STAB1 | 23166 | 0.825 | + | 9 | 0 | 0 |
| CCL18 | 6362 | 0.982 | + | 7 | 0 | 0 |
| SDS | 10993 | 0.773 | + | 9 | 0 | 0 |
| GIMAP5 | 55340 | 1.005 | + | 7 | 0 | 0 |
| CSF1R | 1436 | 1.023 | + | 9 | 0 | 0 |
| VAMP5 | 10791 | 0.895 | + | 9 | 0 | 0 |
| ADAP2 | 55803 | 0.929 | + | 8 | 0 | 0 |
| FLVCR2 | 55640 | 0.834 | + | 9 | 0 | 0 |
| GIMAP2 | 26157 | 0.822 | + | 8 | 0 | 0 |
| HLA-G | 3135 | 0.839 | + | 6 | 0 | 0 |
| CAPG | 822 | 0.874 | + | 9 | 0 | 0 |
| CD247 | 919 | 0.734 | + | 9 | 0 | 0 |
| FOXN2 | 3344 | 0.86 | + | 9 | 0 | 0 |
| EMILIN2 | 84034 | 0.787 | + | 9 | 0 | 0 |
| GIMAP8 | 155038 | 0.952 | + | 9 | 0 | 0 |
| CD80 | 941 | 1.003 | + | 8 | 0 | 0 |
| TRPV2 | 51393 | 0.843 | + | 9 | 0 | 0 |
| HK3 | 3101 | 0.911 | + | 9 | 0 | 0 |
| LPAR1 | 1902 | 0.772 | + | 9 | 0 | 0 |
| C1QA | 712 | 0.989 | + | 9 | 0 | 0 |
| MAP1S | 55201 | 0.823 | + | 9 | 0 | 0 |
| SLAMF8 | 56833 | 1.037 | + | 8 | 0 | 0 |
| H4C8 | 8365 | 1.017 | + | 7 | 0 | 0 |
| CKAP4 | 10970 | 0.719 | + | 9 | 0 | 0 |
| PHF11 | 51131 | 0.946 | + | 9 | 0 | 0 |
| AIP | 9049 | 0.903 | + | 9 | 0 | 0 |
| GTPBP2 | 54676 | 1.045 | + | 9 | 0 | 0 |
| DUSP3 | 1845 | 0.959 | + | 9 | 0 | 0 |
| GZMH | 2999 | 0.874 | + | 7 | 0 | 0 |
| RUBCN | 9711 | 0.852 | + | 9 | 0 | 0 |
| CDKN1C | 1028 | 0.851 | + | 9 | 0 | 0 |
| MFSD13A | 79847 | 0.86 | + | 8 | 0 | 0 |
| HLA-B | 3106 | 0.728 | + | 9 | 0 | 0 |
| SCARB2 | 950 | 0.745 | + | 9 | 0 | 0 |
| LRRC8C | 84230 | 0.654 | + | 9 | 0 | 0 |
| NKG7 | 4818 | 1.123 | + | 9 | 0 | 0 |
| STAT4 | 6775 | 0.825 | + | 9 | 0 | 0 |
| SH2D1A | 4068 | 0.653 | + | 7 | 0 | 0 |
| MYEOV | 26579 | 0.835 | + | 9 | 0 | 0 |
| SLFN12 | 55106 | 0.772 | + | 8 | 0 | 0 |
| AOAH | 313 | 0.785 | + | 9 | 0 | 0 |
| NOD1 | 10392 | 0.813 | + | 8 | 0 | 0 |
| OLR1 | 4973 | 0.731 | + | 9 | 0 | 0 |
| MAD2L2 | 10459 | 0.737 | + | 9 | 0 | 0 |
| RNASE2 | 6036 | 1.057 | + | 7 | 0 | 0 |
| DEFB1 | 1672 | 1.007 | + | 8 | 0 | 0 |
| CMKLR1 | 1240 | 1.011 | + | 9 | 0 | 0 |
| VASH1 | 22846 | 0.698 | + | 9 | 0 | 0 |
| UBE2F | 140739 | 1.016 | + | 8 | 0 | 0 |
| TNS3 | 64759 | 0.716 | + | 9 | 0 | 0 |
| TSPAN14 | 81619 | 0.773 | + | 9 | 0 | 0 |
| GAL3ST4 | 79690 | 0.752 | + | 8 | 0 | 0 |
| SLC1A3 | 6507 | 0.848 | + | 8 | 0 | 0 |
| C6orf47 | 57827 | 0.768 | + | 8 | 0 | 0 |
| MGAT1 | 4245 | 0.826 | + | 9 | 0 | 0 |
| SERPINB9P1 | 221756 | 0.784 | + | 6 | 0 | 0 |
| IL2RG | 3561 | 0.72 | + | 9 | 0 | 0 |
| SDSL | 113675 | 0.784 | + | 8 | 0 | 0 |
| RETN | 56729 | 0.704 | + | 6 | 0 | 0 |
| SERTAD1 | 29950 | 0.871 | + | 9 | 0 | 0 |
| GZMK | 3003 | 0.722 | + | 7 | 0 | 0 |
| MS4A4A | 51338 | 0.95 | + | 7 | 0 | 0 |
| TMEM176B | 28959 | 1.012 | + | 7 | 0 | 0 |
| HEG1 | 57493 | 0.868 | + | 9 | 0 | 0 |
| GZMB | 3002 | 1.23 | + | 9 | 0 | 0 |
| PLOD1 | 5351 | 0.689 | + | 9 | 0 | 0 |
| RENBP | 5973 | 0.718 | + | 9 | 0 | 0 |
| ELMO2 | 63916 | 0.784 | + | 9 | 0 | 0 |
| OLFML2B | 25903 | 1.004 | + | 8 | 0 | 0 |
| FAM225A | 286333 | 0.744 | + | 6 | 0 | 0 |
| CD5 | 921 | 0.698 | + | 9 | 0 | 0 |
| MTHFD2 | 10797 | 0.739 | + | 8 | 0 | 0 |
| HLA-A | 3105 | 0.799 | + | 9 | 0 | 0 |
| CD33 | 945 | 0.649 | + | 6 | 0 | 0 |
| MAFB | 9935 | 1.293 | + | 9 | 0 | 0 |
| PRF1 | 5551 | 1.066 | + | 9 | 0 | 0 |
| SMCO4 | 56935 | 0.64 | + | 8 | 0 | 0 |
| CD2 | 914 | 0.652 | + | 9 | 0 | 0 |
| RRAS | 6237 | 0.905 | + | 8 | 0 | 0 |
| CD7 | 924 | 0.912 | + | 9 | 0 | 0 |
| MILR1 | 284021 | 0.935 | + | 8 | 0 | 0 |
| DOK2 | 9046 | 0.703 | + | 8 | 0 | 0 |
| GIMAP7 | 168537 | 0.807 | + | 9 | 0 | 0 |
| TMEM92 | 162461 | 0.916 | + | 8 | 0 | 0 |
| OSCAR | 126014 | 0.841 | + | 7 | 0 | 0 |
| LGALS1 | 3956 | 1.231 | + | 9 | 0 | 0 |
| TNFAIP8L2 | 79626 | 0.627 | + | 6 | 0 | 0 |
| FCGR1BP | 2210 | 0.879 | + | 9 | 0 | 0 |
| RASSF4 | 83937 | 0.858 | + | 9 | 0 | 0 |
| SQOR | 58472 | 1.004 | + | 8 | 0 | 0 |
| NOCT | 25819 | 0.756 | + | 9 | 0 | 0 |
| TICAM1 | 148022 | 0.826 | + | 9 | 0 | 0 |
| ASPHD2 | 57168 | 0.852 | + | 6 | 0 | 0 |
| DESI1 | 27351 | 0.737 | + | 9 | 0 | 0 |
| MFSD12 | 126321 | 0.727 | + | 9 | 0 | 0 |
| FBXO6 | 26270 | 1.307 | + | 9 | 0 | 0 |
| TMEM199 | 147007 | 0.759 | + | 8 | 0 | 0 |
| STOM | 2040 | 0.858 | + | 9 | 0 | 0 |
| APBA3 | 9546 | 0.832 | + | 8 | 0 | 0 |
| CD300LF | 146722 | 0.788 | + | 9 | 0 | 0 |
| P2RX4 | 5025 | 0.788 | + | 9 | 0 | 0 |
| TOR1B | 27348 | 1.111 | + | 9 | 0 | 0 |
| MYO1G | 64005 | 0.906 | + | 9 | 0 | 0 |
| C2 | 717 | 0.906 | + | 9 | 0 | 0 |
| TRIM5 | 85363 | 0.846 | + | 9 | 0 | 0 |
| RIPK3 | 11035 | 0.808 | + | 8 | 0 | 0 |
| TENT5A | 55603 | 0.861 | + | 8 | 0 | 0 |
| HLA-F | 3134 | 0.883 | + | 9 | 0 | 0 |
| ACOD1 | 730249 | 1.185 | + | 7 | 0 | 0 |
| CD68 | 968 | 1.034 | + | 9 | 0 | 0 |
| LY96 | 23643 | 0.719 | + | 9 | 0 | 0 |
| IL32 | 9235 | 0.772 | + | 9 | 0 | 0 |
| BTN3A3 | 10384 | 0.875 | + | 9 | 0 | 0 |
| GZMA | 3001 | 0.938 | + | 9 | 0 | 0 |
| TMUB2 | 79089 | 0.704 | + | 8 | 0 | 0 |
| POLR3D | 661 | 0.834 | + | 9 | 0 | 0 |
| PLA2G7 | 7941 | 0.988 | + | 9 | 0 | 0 |
| IL6 | 3569 | 0.84 | + | 9 | 0 | 0 |
| SLCO2B1 | 11309 | 0.793 | + | 8 | 0 | 0 |
| AIM2 | 9447 | 1.302 | + | 9 | 0 | 0 |
| EMP3 | 2014 | 0.873 | + | 9 | 0 | 0 |
| BATF2 | 116071 | 1.202 | + | 9 | 0 | 0 |
| NUCB1 | 4924 | 0.987 | + | 9 | 0 | 0 |
| ICAM2 | 3384 | 0.76 | + | 7 | 0 | 0 |
| TMEM176A | 55365 | 0.918 | + | 7 | 0 | 0 |
| CCL8 | 6355 | 1.495 | + | 9 | 0 | 0 |
| NEXN | 91624 | 0.937 | + | 9 | 0 | 0 |
| IFI35 | 3430 | 1.352 | + | 9 | 0 | 0 |
| KCTD14 | 65987 | 1.048 | + | 6 | 0 | 0 |
| ABCD1 | 215 | 0.872 | + | 9 | 0 | 0 |
| SOCS1 | 8651 | 0.934 | + | 9 | 0 | 0 |
| DRAM1 | 55332 | 0.911 | + | 9 | 0 | 0 |
| HLA-E | 3133 | 0.931 | + | 9 | 0 | 0 |
| DUSP6 | 1848 | 0.887 | + | 9 | 0 | 0 |
| BST2 | 684 | 1.09 | + | 9 | 0 | 0 |
| MT2A | 4502 | 1.231 | + | 9 | 0 | 0 |
| HESX1 | 8820 | 0.839 | + | 8 | 0 | 0 |
| IFNL2 | 282616 | 0.882 | + | 8 | 0 | 0 |
| GRAMD1B | 57476 | 0.963 | + | 9 | 0 | 0 |
| APOBEC3G | 60489 | 0.715 | + | 9 | 0 | 0 |
| TLR7 | 51284 | 0.846 | + | 8 | 0 | 0 |
| IL15RA | 3601 | 0.821 | + | 8 | 0 | 0 |
| COLGALT1 | 79709 | 0.777 | + | 9 | 0 | 0 |
| LAIR1 | 3903 | 0.963 | + | 9 | 0 | 0 |
| CCL2 | 6347 | 1.283 | + | 9 | 0 | 0 |
| IL27 | 246778 | 0.711 | + | 8 | 0 | 0 |
| PPP1R3D | 5509 | 0.798 | + | 9 | 0 | 0 |
| HAMP | 57817 | 1.028 | + | 8 | 0 | 0 |
| GBP1P1 | 400759 | 1.097 | + | 7 | 0 | 0 |
| TMEM140 | 55281 | 0.882 | + | 9 | 0 | 0 |
| EDEM2 | 55741 | 0.813 | + | 8 | 0 | 0 |
| GIMAP6 | 474344 | 0.988 | + | 9 | 0 | 0 |
| CALHM6 | 441168 | 1.203 | + | 7 | 0 | 0 |
| TRIM21 | 6737 | 0.908 | + | 9 | 0 | 0 |
| ATF5 | 22809 | 0.737 | + | 9 | 0 | 0 |
| KLHDC7B | 113730 | 0.885 | + | 9 | 0 | 0 |
| RTP4 | 64108 | 1.2 | + | 9 | 0 | 0 |
| RBCK1 | 10616 | 0.878 | + | 9 | 0 | 0 |
| PARP10 | 84875 | 0.76 | + | 9 | 0 | 0 |
| ETV7 | 51513 | 1.031 | + | 9 | 0 | 0 |
| TDRD7 | 23424 | 0.817 | + | 9 | 0 | 0 |
| SHFL | 55337 | 1.404 | + | 7 | 0 | 0 |
| NOA1 | 84273 | -0.876 | - | 8 | 0 | 0 |
| CLDN8 | 9073 | -0.742 | - | 7 | 0 | 0 |
| CCDC190 | 339512 | -0.941 | - | 9 | 0 | 0 |
| PAPOLA-DT | 730202 | -1.047 | - | 5 | 0 | 0 |
| MPC2 | 25874 | -0.714 | - | 9 | 0 | 0 |
| EBNA1BP2 | 10969 | -0.775 | - | 9 | 0 | 0 |
| SMIM19 | 114926 | -0.743 | - | 9 | 0 | 0 |
| ALDH9A1 | 223 | -0.758 | - | 9 | 0 | 0 |
| VDAC3 | 7419 | -0.713 | - | 9 | 0 | 0 |
| PPP4R3B | 57223 | -0.838 | - | 9 | 0 | 0 |
| DUS4L | 11062 | -0.683 | - | 7 | 0 | 0 |
| SGSM2 | 9905 | -0.641 | - | 9 | 0 | 0 |
| COQ3 | 51805 | -0.689 | - | 7 | 0 | 0 |
| EEF1G | 1937 | -0.657 | - | 9 | 0 | 0 |
| LOC541473 | 541473 | -0.787 | - | 6 | 0 | 0 |
| CCT6B | 10693 | -1.025 | - | 8 | 0 | 0 |
| TSTD1 | 100131187 | -0.68 | - | 8 | 0 | 0 |
| TMEM14B | 81853 | -0.768 | - | 8 | 0 | 0 |
| ERCC1 | 2067 | -0.702 | - | 9 | 0 | 0 |
| PEBP1 | 5037 | -1.035 | - | 9 | 0 | 0 |
| CAT | 847 | -0.764 | - | 9 | 0 | 0 |
| QARS1 | 5859 | -0.952 | - | 8 | 0 | 0 |
| PNMA1 | 9240 | -0.726 | - | 9 | 0 | 0 |
| DDX46 | 9879 | -0.711 | - | 9 | 0 | 0 |
| HACL1 | 26061 | -0.897 | - | 8 | 0 | 0 |
| DMKN | 93099 | -0.936 | - | 9 | 0 | 0 |
| FAM174A | 345757 | -0.713 | - | 9 | 0 | 0 |
| ANKRD6 | 22881 | -0.759 | - | 9 | 0 | 0 |
| COQ7 | 10229 | -0.724 | - | 9 | 0 | 0 |
| PER3 | 8863 | -0.679 | - | 9 | 0 | 0 |
| INPP5E | 56623 | -0.8 | - | 9 | 0 | 0 |
| TRIM45 | 80263 | -0.74 | - | 6 | 0 | 0 |
